# How many leaders does it take to lead a sports team? The relationship between the number of leaders and the effectiveness of professional sports teams

**DOI:** 10.1371/journal.pone.0218167

**Published:** 2019-06-10

**Authors:** Francisco M. Leo, Tomás García-Calvo, Inmaculada González-Ponce, Juan J. Pulido, Katrien Fransen

**Affiliations:** 1 Department of Didactics of Musical, Plastic and Corporal Expression, Faculty of Teacher Training, University of Extremadura, Cáceres, Extremadura, Spain; 2 Department of Didactics of Musical, Plastic and Corporal Expression, Faculty of Sports Sciences, University of Extremadura, Cáceres, Extremadura, Spain; 3 Department of Psychology and Anthropology, Faculty of Education, University of Extremadura, Badajoz, Extremadura, Spain; 4 Department of Sports and Health, Faculty of Human Kinetics, University of Lisbon, Lisboa, Portugal; 5 Department of Movement Sciences, Faculty of Movement and Rehabilitation Sciences, KU Leuven, Leuven, Belgium; Instituto Politecnico de Viana do Castelo, PORTUGAL

## Abstract

This study aimed to analyze the number of task, social and external athlete leaders within sports teams, and to examine the effectiveness of different leadership structures in male and female teams. The participants were 317 male and 214 female soccer players belonging to 18 teams playing in the third highest male division and to 13 teams playing in the highest female division in Spain, respectively. First, we identified the leadership structure in each team (i.e., having zero, one, two or three leaders); second, we grouped the teams according to these leadership structures; and third, MANOVA was used to compare different leadership groups in terms of their effectiveness. The results demonstrated that: (a) the most common structure within the teams was to have one task leader, one social leader, and two external leaders; (b) shared leadership across and within leadership roles was seen as the most effective leadership structure for male and female teams; and (c) male teams showed more benefits when having more task and external leaders, while female teams experienced more benefits when having more task and social leaders on the team. Based on these findings, coaches can optimize their team’s functioning by implementing a structure of shared leadership within their teams, both across and within the different leadership roles.

## Introduction

Leadership is an interactive process between leader and followers, where the leader tries to guide and influence a group of individuals toward common goals [1]. Leadership can be seen as effective when a leader succeeds in creating a good team atmosphere, strengthening the team’s cohesion and communication, and establishing a strong work ethic [2]. This effective leadership is, in turn, an important driver of the team’s functioning and effectiveness [3, 4]; that is, the team’s ability to develop adequate cognitive, motivational, affective and coordinative processes [5].

Traditionally, leadership research has focused on the coach as formal leader of the team, guiding the team to optimal performance from a top-down, hierarchical perspective [6, 7]. However, leadership is not restrictive to the coach; players within the team can also take on important leadership roles, which is a phenomenon known as athlete leadership. Athlete leadership has been defined as “an athlete occupying a formal or informal role within a team, who influences a group of members to achieve a common goal” [8]. By taking on leadership roles, athlete leaders can exert significant influence on the group. To illustrate this, previous research has demonstrated how high-quality leaders fostered the intrinsic motivation of their teammates [9], strengthened their team confidence and their team identification [10], while also instigating cohesion within the team [11], and ultimately improving team performance [12]. In short, leadership within the team is considered an important driver of sports success, or, in other words, of the team’s ability to achieve its goals [5].

### Athlete leadership

As the above definition of athlete leadership by Loughead et al. [8] highlighted, two types of athlete leaders can be identified based on their formal recognition as leaders—formal and informal leaders. A player occupies a formal role when he or she is formally recognized as a leader (e.g., captain or assistant captain). In contrast, players occupy informal roles when they do not have that formal recognition, but instead have gained their leadership status through social interactions recognizing their capacity to influence their teammates [8]. Moving away from the traditional focus on the team captain [13, 14], recent findings have pointed out that, in most sports teams, informal leaders are often perceived as better leaders than the formal team captain [10, 15]. To illustrate this, a study developed by Fransen, Vanbeselaere et al. [15], analyzing 4,451 players and coaches in nine team sports, noted that in only 1% of the teams did the team captain fulfill all leadership roles. It is thus the informal leaders who are often perceived as better leaders than the team captain.

In addition to the formal-informal leader distinction, leaders can also be categorized in terms of their function. Within their role differentiation theory, Bales et al. [16] distinguished two leadership functions that individuals can fulfill, namely task functions (i.e., making decisions, responding to or resolving adverse situations, and establishing tactical instructions during matches that help the team achieve its goals and objectives) and social functions (i.e., establishing good relationships among peers, serving as a trusted person, and mediating in socially controversial situations in the group that will help satisfy the psychosocial needs of all team members). Loughead et al. [8] further extended this role categorization by adding a third role to the role of task and social leader, namely the role of external leader (i.e., being a team representative at club meetings, press conferences, and sponsors to help establish appropriate relationships with the team environment). The fulfillment of these three types of leadership has several benefits, as it promotes greater identification of players with the team and greater confidence in the team [17], a stronger task and social cohesion [11], and more optimal team functioning, resulting ultimately in a better team performance [12].

### Different leadership structures

The above findings seem to suggest that, when coaches share their leadership responsibilities with their players, this structure of shared leadership maximizes chances of team success. These findings are in line with earlier findings in the organizational setting, demonstrating that shared leadership explains a unique variance in team effectiveness (i.e., behavioral processes and emergent states, attitudes, perceived performance and objective performance) over and above that of vertical leadership [2, 18]. However, there is still a knowledge gap when it comes to what degree of shared leadership is optimal. In fact, shared leadership covers a broad continuum, ranging from the coach sharing the leadership with one leader (e.g., team captain) to the situation in which all athletes occupy a leadership role. Furthermore, leadership can be shared across leadership roles (i.e., where different people occupy the three leadership roles) but also within each leadership role (e.g., having more than one task leader).

To analyze the current situation in sports teams, our first aim was to analyze the number of leaders within sports teams in each of the three leadership roles (i.e., task, social, and external leader). This will allow us to determine the athlete leadership structure that exists in sports teams [6, 8]. Evidence supporting this aim was found by Loughead et al. [8], who revealed in this respect that, on average, 15% of all players in a team are perceived as task leaders, 11% as social leaders, and 8% as external leaders. Fransen et al. [15] found in their study that the number of external leaders was lower than the number of task and social leaders. Furthermore, Fransen [19] examined the number of leaders within a specific role. Her findings revealed that teams had more task leaders than social or external leaders. But given that her sample was so small, no reliable conclusions could be made. In the present study we wanted to replicate these findings by examining a larger sample within soccer. Given the lack of previous reliable evidence on the exact number of leaders in a team on the different leadership roles, we decided not to formulate an a priori hypothesis.

### Shared leadership and team effectiveness

More important than the number of leaders in each leadership role is knowing what the most effective leadership structure is. Our next research aim is thus to achieve more insight about the optimal number of leaders in each role. We will ground our hypotheses on the findings of two previous studies examining this question, relating the number of leaders with variables associated with better team effectiveness (i.e., behavioral processes, attitudes, and perceived performance). First, Eys et al. [20] identified the optimal number of leaders in university teams. Their results suggested that, when teams had an equal number of athlete leaders in each of the three leadership roles (regardless of how many leaders fulfilled the role), athletes in these teams rated higher satisfaction with their integration in the team and with the team’s performance. A second study was conducted by Fransen [19] in a sample of 267 athletes playing on soccer, volleyball, and basketball teams. The author examined the relationship between the number of leaders in the different leadership roles and two team-level variables related to team effectiveness, namely task and social cohesion [2]. The results revealed that the greater the number of leaders in each leadership role, the better the task and social cohesion within the team.

Given the contrasting findings of the two studies, our aim is to test this research question in a larger sample, while also including a broad variety of outcome variables, both at the individual level (i.e., role clarity, role conflict, intention to continue) and at the team level (i.e., cohesion, team conflict, collective efficacy, team performance). These variables were selected because they allowed us to assess the different types of team effectiveness identified by Wang et al. [2], namely (a) behavioral processes (i.e., role clarity, role conflict, cohesion, team conflict, and collective efficacy), (b) attitudes (i.e., intention to continue), and (c) perceived performance. Also, previous research indicated a close relationship of the above variables with performance [21–25]. More specifically, with respect to behavioral processes, it has been shown that, if team players have clear and coherent information about their role (i.e., role clarity), if they are strongly united as team (i.e., team cohesion), and if they believe in the team’s abilities (i.e., collective efficacy), their performance is likely to improve [21–26]. In contrast, when team members receive incongruent information about their roles (i.e., role conflict) or think that their interests are being thwarted by other players (i.e., team conflict), their performance is likely to deteriorate [27–29]. Second, regarding attitudes, when players intend to continue playing on the same team the next year, this may be an indicator that they are satisfied with personal and team performance [30]. Furthermore, perceived performance is one of the parameters most widely used to assess team effectiveness [31]. Whereas objective performance measures have the disadvantage of being influenced by many confounding factors (e.g., the team’s inherent abilities, the opponent’s strength, the weather and other environmental factors, the referee’s decision quality, etc.), perceived performance measures might provide a more reliable measure of performance [32].

It is important to note that previous studies have focused solely on positive outcomes. However, it is essential for a team with an optimal leadership structure to also be able to overcome potential barriers to team success such as team conflict and role conflict [27–29]. For example, although having more than one task leader could lead to a stronger confidence amongst members [15], this is only beneficial when it is not counteracted by a larger team conflict [33]. Therefore, in the current study, we will address previous suggestions of, for example, Marks et al. [34] and analyze how the leadership structure in the team (i.e., the number of leaders in each of the roles) is related to the conflicts experienced in the team, as well as to the conflict associated with role occupancy.

*H1*: *Given the previous established benefits of having shared leadership within each leadership role* [19], *we expect that a higher number of leaders within a team will be positively related to indicators of team effectiveness (role clarity*, *team cohesion*, *collective efficacy*, *intention to continue and perceived team performance)*, *and negatively related to team conflict and role conflict*. *We expect this hypothesis to hold for task leadership (H1a)*, *social leadership (H1b)*, *and external leadership (H1c)*.

*H2*: *When looking at the leadership structure across the different leadership roles*, *we expect to find that a balanced number of leaders in each role is the most favorable leadership structure* [20].

### Differences between male and female teams

Previous studies failed to provide more insight about whether the ideal leadership structure differs for male and female teams. Gender differences have been demonstrated, however, with respect to leader characteristics. For example, Fransen et al. [35] found that female leaders were more likely to occupy a central position (i.e., having significant interaction with other players) than male leaders in field hockey. Moran and Weiss [36] added that female leaders were characterized by ability variables, while male leaders were characterized by psychosocial and ability variables. However, other studies did not find these gender differences when identifying leaders’ characteristics [37].

Also with respect to group dynamics, researchers have found differences in male and female teams [22–24]. For example, the relationship between cohesion and performance was observed to be significantly larger for females than for males [24]. It should be noted that, for the other variables of interest, no gender differences have yet been investigated. As Leo et al. [33] suggested, it would be interesting to achieve a better insight about how these group processes, and more specifically the benefits of a particular athlete leadership structure, might differ for male and female athletes. Given the lack of previous evidence of potential differences with respect to the ideal leadership structure in male and female teams, no specific hypothesis for this third research aim can be formulated a priori.

## Materials and methods

### Procedure

We invited all soccer clubs of the highest female division (16 teams) and the third highest male division (20 teams) in Spain to participate in our study and informed them via their club management or coach about the objectives and procedures of our research. In the 31 teams that agreed to participate, all players were informed about the research objectives, and they were told that their participation was voluntary and the provided answers would be treated confidentially. A cross-sectional design was used and a single data collection assessment was completed near the end of the competitive period to ensure that the players would have gained adequate insight about the informal leadership qualities of their fellow team members. Participants completed the questionnaire individually in the locker room in the absence of their coach. The process took approximately 30 minutes. The principal investigator was present during this time to answer any questions that arose during the process. The study received ethical approval from the first author´s university; Vice-Rectorate of Research, Transfer and Innovation—Delegation of the Bioethics and Biosafety Commission (Protocol number: 137/2015). All participants were treated according to the American Psychological Association ethical guidelines regarding consent, confidentiality, and anonymity of responses.

### Participants

The participants were 531 professional soccer players in the Spanish Soccer League. The sample included 317 male players (18 teams playing in the third highest division) and 214 female players (13 teams playing in the highest division). The male players were between 17 and 37 years old (*M* = 25.25; *SD* = 4.70) with an average soccer experience of 15.98 years (*SD* = 5.53), whereas the female players were between 16 and 36 years old (*M* = 22.22, *SD* = 4.41) with an average soccer experience of 12.27 years (*SD* = 3.96). From the original sample of 559 questionnaires collected at the end of the season, 28 questionnaires (i.e., 5.01%) were excluded due to invalid completion (i.e., more than 50% of the questions were not completed, more than one answer was given to the same question, or the presence of a clear response pattern).

### Measures

#### Athlete leadership

To identify those individuals on the team who were perceived to be task, social, and external athlete leaders, participants responded to three open-ended questions, in line with previous guidelines of Loughead et al. [8]. More specifically, participants were asked to “List the names of team members (including yourself if applicable) that, according to you, most strongly contribute to your team’s task/social/external factors. That is, please list team members who do or have done at least one, some, or all of the following actions”. Following these instructions, a list of behavioral characteristics of task, social, and external leadership was provided to give the participants a frame of reference similar to the one suggested by Kogler Hill [38] and Eys et al. [20]. These behavioral characteristics are presented in Table 1.

**Table 1:** The behavioral characteristics of the different leaders.

Behavioral characteristics of leaders		
Task leaders	Social leaders	External leaders
− helping to focus the team on its goals	− contributing to team harmony	− promoting the team within the community
− helping to clarify responsibilities for teammates	− ensuring teammates are involved and included in team events	− representing the team’s interests in meetings with coaching staff or league organizers
− assisting in decision making	− helping to solve interpersonal conflicts that may arise within the team	− attempting to secure necessary or desired resources, support, and recognition for the team
− offering instruction to teammates when required	− offering support and being trusted by teammates	− buffering team members from outside distractions
− helping the team to perform to the best of its ability	− treating team members in a fair and consistent manner	− sharing relevant external information with the team

In the first step, we calculated the number of times each player was cited by his/her team members as being a task, social, or external leader. In the next step, we followed the suggestions of Loughead et al. [8] and classified leaders as such if at least half of their team members who responded to the questionnaire endorsed them as a task, social, or external leader. This method has been used extensively in previous studies to assess the number of athlete leaders [11, 15, 17, 19, 20].

Subsequently, for each of the leadership roles, we distinguished three possible leadership structures, namely (a) the leadership role is occupied by no one (zero leaders); (b) the leadership role is occupied by a single player (one leader); and (c) the leadership role is occupied by multiple players. Although the last category originally included a broader range of possible leadership structures, ranging from having two leaders to having all the players on the team occupying that leadership role, in reality, there were no teams with more than three leaders in a specific leadership role.

#### Role clarity

To assess role clarity, we used the 12-item Spanish version [39] of the Role Ambiguity Scale [21]. An example of role clarity includes “I am clear about the different responsibilities that make up my role.” Players responded to all items on a 9-point scale ranging from 1 (*strongly disagree*) to 9 (*strongly agree*). Thus, higher ratings of agreement indicated greater role clarity and, hence, less role ambiguity.

#### Role conflict

To assess role conflict, we used a 6-item scale developed by Beauchamp and Bray [27]. An example item is “I sometimes receive conflicting information of what my role is.” Players rated these items on a 5-point scale ranging from 1 (*strongly disagree*) to 5 (*strongly agree*). Thus, higher ratings of agreement indicated greater role conflict.

#### Team conflict

To assess team conflict, we used the 6-item scale developed by Tekleab et al. [29] following the guidelines from Jehn [40]. This instrument has six items that comprise two factors: Task Conflict (three items, e.g., “How frequently did people on your team disagree regarding the work being done?”) and Relationship Conflict (three items, e.g., “How frequently were personality conflicts present on your team?”). Players responded to all items on a 9-point scale ranging from 1 (*never*) to 9 (*always*).

#### Team cohesion

The Short Spanish version of the GEQ [41] developed by Leo et al. [42] was used to assess team cohesion. This 12-item inventory comprises two main factors, namely Task Cohesion (6 items, e.g., “Team members are united in their efforts to reach their performance goals in training sessions and games”) and Social Cohesion (6 items, e.g., “Team members would like to spend time together in situations other than training and games”). Responses were made on a 9-point scale ranging from 1 (*strongly disagree*) to 9 (*strongly agree*).

#### Collective efficacy

To assess collective efficacy, The Football Collective Efficacy Questionnaire [43] was used. This 26-item instrument starts with the stem “Our team has confidence in our capability to…” The items refer to specific football situations (e.g., resolving game situations in the attacking phase). Responses were made on a 5-point scale ranging from 1 (*bad*) to 5 (*excellent*).

#### Intention to continue

To value each player’s choice of remaining with the same team, coach or teammates, the players responded to three questions [30]; “Would you like to continue next year (1) on the same team?; (2) … with the same coach?; and (3) …with the same teammates?” Responses were made on a 5-point scale ranging from 1 (*strongly disagree*) to 5 (*strongly agree*).

#### Perceived team performance

To assess perceived team performance, we assessed the players’ subjective perception through a single-item scale, which was previously used by Dithurbide et al. [44]. More specifically, players were asked to rate their team’s performance on a 5-point scale ranging from 1 (*poor*) to 5 (*excellent*). Although, single-item scales have been criticized, some authors have argue that this type of measure has greater ecological validity [45, 46].

### Data analysis

The obtained data were analyzed with SPSS 19.0. Means, standard deviations, intraclass correlation coefficients, and bivariate correlations among variables under investigation were calculated to meet the first aim. Intraclass correlation coefficients for the different subscales ranged between .08 and .58, indicating the need for multilevel analysis for comparisons between groups to meet the second, third and fourth aims. Therefore, we followed the recommendations of Heck et al. [47] to calculate multivariate multilevel models for correlated outcome variables with mixed models (see chapter 7, pp. 247–268). We performed one model for each research question. The models were built to examine the differences in outcome variables for teams having different leadership structures (i.e., zero, one, two, or three task leaders) with fixed effects to control differences between athletes and with random effects to calculate variances for outcome variables and covariance between outcome variables [48]. First, we created a variable to include all the outcomes and included it as dependent variable. We also created a categorical variable (called *index*) to identify each outcome variable (coded from 1 to 9). Secondly, we described *leadership groups* (e.g., task leader), *index*, and leadership groups * index interaction as fixed effects. Thirdly, we included the random effects of *index* at the team level. The model showed instability at the team level (i.e., random effects), and the parameter of covariance, the test statistic, and the confidence interval could not be calculated. This is likely due to the small sample size for the second level (i.e., 31 teams) and therefore, the model tends to encounter convergence problems, as the variance for the between-level parameters tends to be small [42]. As a result, for comparisons between different groups (zero, one, two, or three leaders), Multivariate Analysis of Variance (MANOVA) was used with subsequent Bonferroni post-hoc analyses to simplify the models. Furthermore, Bonferroni adjustments for multiple comparisons were used in all cases. It should be noted that the results obtained in the fixed effects of the mixed models and in the MANOVA were similar.

## Results

### Preliminary analysis

We ran a confirmatory factor analysis (CFA) for each scale. Data offered support for the factor structure of the variables under investigation, showing acceptable model fit for role clarity (*χ*^*2*^ = 149.434; *df* = 51; *p* < .001; *CFI* = .96; *TLI* = .95; *RMSEA* = .05; *SRMR* = .02), role conflict (*χ*^*2*^ = 19.75; *df* = 9; *p* < .001; *CFI* = .98; *TLI* = .98; *RMSEA* = .04; *SRMR* = .02), team conflict (*χ*^*2*^ = 129.70; *df* = 48; *p* < .001; *CFI* = .97; *TLI* = .96; *RMSEA* = .05; *SRMR* = .03), collective efficacy (*χ*^*2*^ = 19.07; *df* = 9; *p* = .02; *CFI* = .98; *TLI* = .98; *RMSEA* = .04; *SRMR* = .02), and intention to continue (*χ*^*2*^ = 7.25; *df* = 2; *p* = .007; *CFI* = .97; *TLI* = .90; *RMSEA* = .10; *SRMR* = .03). Furthermore, all scales had acceptable internal consistency (*α* < .70) [49], with the exception of intention to continue, which showed lower values (*α* = .66; see Table 2). Although this value refers to a relatively low internal consistency, Lowenthal [50] recommended that values above .60 should be considered suitable if there is good evidence of validity, if there is good theoretical support for the scale, and if the number of items is less than 10. Given that the present scale meets all these criteria, we deemed the internal consistency as acceptable.

**Table 2:** Descriptive statistics, correlations, and Cronbach’s alpha of all variables of the study.

	*M (SD)*	ICC	1	2	3	4	5	6	7	8	9	10	11
1. Task leaders	1.25 (.76)	-	-										
2. Social leaders	1.07 (.72)	-	.31***	-									
3. External leaders	1.58 (.60)	-	.04	.16***	-								
4. Role clarity	7.42 (1.32)	.08	.06	-.02	-.04	.*95*							
5. Role conflict	2.16 (.84)	.11	-.06	.02	.06	-.41***	.*85*						
6. Relationship conflict	2.61 (1.40)	.40	-.13**	.03	.05	-.34***	.50***	.*90*					
7. Task conflict	3.21 (1.36)	.33	-.29***	-.05	-.02	-.30***	.28***	.54***	.*82*				
8. Social cohesion	6.86 (1.67)	.25	.15**	.05	.08	.52***	-.43***	-.49***	-.48***	.*88*			
9. Task cohesion	6.60 (1.61)	.23	.05	.08	.21***	.35***	-.20***	-.22***	-.40***	.66***	.*84*		
10. Collective efficacy	3.52 (.69)	.35	.20***	.02	-.05	.34***	-.34***	-.49***	-.47***	.63***	.39***	.*84*	
11. Intention to continue	3.70 (.48)	.20	.11**	.10*	12**	34***	-.41***	-.44***	-.36***	.57***	.43***	-39***	.*66*
12. Perceived performance	3.56 (1.04)	.58	.31***	-.10*	-.09*	.23***	-.26***	-.36***	-.51***	.53***	.34***	.54***	40***

*p < .05

**p < .01

***p < .001.

ICC = Intraclass Correlation Coefficient. Cronbach’s alphas are presented in italics on the diagonal.

### Descriptive statistics

Table 2 presents the means, standard deviations, bivariate correlations, and Cronbach’s alpha coefficients for each variable. All scales showed acceptable internal consistency. In general, participants obtained scores above the mid-point of the scale for role clarity, task conflict, task and social cohesion, collective efficacy, intention to continue, and perceived team performance. Participants also obtained scores for role conflict and relationship conflict that were close to the mid-point of the scale. With respect to the correlations, it is interesting to note that the correlations between the different leadership roles (i.e., task, social, and external) were relatively low (i.e., ranging between .04 and .31).

### Most common leadership structure

Table 3 presents the leadership structure for each of the different teams, and more specifically, reveals the number of teams having zero, one, two, or three leaders in each of the three leadership roles. The majority of the teams had one task leader and one social leader. However, with respect to external leaders, most teams had two external leaders on the team.

**Table 3:** The number of teams with zero, one, two, or three leaders.

Task Leaders	0 leaders	1 leader	2 leaders	3 leaders
Overall	5 teams	**15 teams**	10 teams	1 team
Male teams	3 teams	**9 teams**	6 teams	0 teams
Female teams	2 teams	**6 teams**	4 teams	1 team
Social Leaders				
Overall	7 teams	**16 teams**	8 teams	0 teams
Male teams	3 teams	**10 teams**	5 teams	0 teams
Female teams	4 teams	**6 teams**	3 teams	0 teams
External Leaders				
Overall	2 teams	10 teams	**19 teams**	0 teams
Male teams	1 team	8 teams	**9 teams**	0 teams
Female teams	1 team	2 teams	**10 teams**	0 teams

Higher values are presented in bold.

### Most effective leadership structure

The second aim of this study was to analyze the relationship between the numbers of leaders in each leadership role and the team’s effectiveness. For each leadership role, a MANOVA was used to examine the differences between having zero leaders in that role, having one leader in that role, and having multiple leaders in that role.

#### Task leadership

Table 4 presents the different outcomes for each of the different leadership structures (i.e., when having zero, one, two, or three task leaders). The MANOVA yielded a significant difference in all outcome variables depending on the number of leaders (Wilk’s Λ = .77, F_(27,1495.95)_ = 5.04, *p* < .001; partial η^2^ = .08), with the highest scores being found in teams with three task leaders. Only for role clarity and social cohesion, no significant differences were found between teams with zero, one, two, or three task leaders, although a trend in the expected direction was noted.

**Table 4:** Differences between zero, one, two, or three task leaders.

	0 leaders	1 leader	2 leader	3 leaders	*F*	*Differences between number of leaders (p)*					
	*M (SD)*	*M (SD)*	*M (SD)*	*M (SD)*		0–1	0–2	0–3	1–2	1–3	2–3
All teams	(*n* = 86)	(n = 248)	(*n* = 177)	(*n* = 20)							
Role clarity	7.34 (1.39)	7.40 (1.28)	7.41 (1.38)	**8.16 (.67)**	2.24	1.00	1.00	.08	1.00	.07	.09
Role conflict	2.19 (.84)	2.18 (.84)	2.19 (.85)	**1.57 (.52)**	3.52^*^	1.00	1.00	**.02**	1.00	**.01**	**.01**
Task conflict	3.38 (1.25)	3.23 (1.40)	3.32 (1.30)	**1.32 (.38)**	14.50^***^	1.00	1.00	**< .001**	1.00	**< .001**	**< .001**
Relationship conflict	3.14 (1.32)	2.79 (1.52)	2.28 (1.14)	**1.17 (.28)**	17.23^***^	.22	**< .001**	**< .001**	**.001**	**< .001**	**.003**
Task cohesion	6.39 (1.56)	6.47 (1.75)	6.70 (1.42)	**8.13 (.79)**	7.47^***^	1.00	.75	**< .001**	.90	**< .001**	**.001**
Social cohesion	6.70 (1.46)	6.90 (1.77)	6.81 (1.64)	**7.60 (1.28)**	1.73	1.00	1.00	.17	1.00	.44	.27
Collective efficacy	3.42 (.61)	3.41 (.71)	3.63 (.65)	**4.22 (.41)**	11.62^***^	1.00	.08	**< .001**	**.010**	**< .001**	**.001**
Intention to continue	3.63 (.92)	3.65 (.98)	3.69 (.94)	**4.68 (.46)**	7.71^***^	1.00	1.00	**< .001**	1.00	**< .001**	**< .001**
Perceived performance	3.29 (.82)	3.33 (1.16)	3.88 (.79)	**4.80 (.41)**	23.88^***^	1.00	**< .001**	**< .001**	**< .001**	**< .001**	**< .001**
Male teams	(*n* = 55)	(*n* = 156)	(*n* = 106)	(*n* = 0)							
Role clarity	7.44 (1.42)	**7.61 (1.17)**	7.22 (1.55)		2.64	1.00	.97		.06		
Role conflict	**2.09 (.85)**	2.11 (.82)	2.27 (.83)		1.61	1.00	.60		.39		
Task conflict	3.03 (1.22)	**3.00 (1.37)**	3.57 (1.27)		6.53^**^	1.00	**.04**		**.002**		
Relationship conflict	3.13 (1.36)	2.51 (1.55)	**2.32 (1.20)**		5.90^**^	**.02**	**.002**		.87		
Task cohesion	**6.71 (1.48)**	6.69 (1.71)	6.60 (1.40)		.11	1.00	1.00		1.00		
Social cohesion	6.83 (1.46)	6.92 (1.68)	**6.61 (1.56)**		1.17	1.00	1.00		.35		
Collective efficacy	3.61 (.52)	**3.56 (.66)**	3.62 (.61)		.31	1.00	1.00		1.00		
Intention to continue	3.55 (.99)	3.70 (.98)	**3.80 (.89)**		1.25	1.00	.23		.73		
Perceived performance	3.29 (.87)	3.51 (1.22)	**3.92 (.66)**		8.94^***^	.48	**.001**		**.005**		
Female teams	(*n* = 31)	(*n* = 92)	(*n* = 71)	(*n* = 20)							
Role clarity	7.16 (1.33)	7.04 (1.37)	7.68 (1.03)	**8.16 (.67)**	7.01^***^	1.00	.28	**.02**	**.005**	**.001**	.740
Role conflict	2.37 (.81)	2.28 (.86)	2.06 (.87)	**1.57 (.52)**	4.73^**^	1.00	.56	**.006**	.60	**.004**	.12
Task conflict	3.99 (1.06)	3.62 (1.36)	2.94 (1.25)	**1.32 (.38)**	24.00^***^	.93	**.001**	**< .001**	**.004**	**< .001**	**< .001**
Relationship conflict	3.16 (1.26)	3.25 (1.09)	2.20 (1.03)	**1.17 (.27)**	23.87^***^	1.00	**.002**	**< .001**	**< .001**	**< .001**	**.004**
Task cohesion	5.83 (1.55)	6.09 (1.77)	6.86 (1.45)	**8.13 (.78)**	12.40^***^	1.00	**.01**	**< .001**	**.01**	**< .001**	**.009**
Social cohesion	6.47 (1.47)	6.84 (1.92)	7.12 (.1.73)	**7.60 (1.28)**	2.06	1.00	.50	.15	1.00	.48	1.00
Collective efficacy	3.06 (.61)	3.17 (.73)	3.63 (.72)	**4.22 (.41)**	17.58^***^	1.00	**.001**	**< .001**	**< .001**	**< .001**	**.006**
Intention to continue	3.33 (.98)	3.61 (.97)	3.94 (.99)	**4.68 (.46)**	9.73^***^	.94	**.02**	**< .001**	.18	**< .001**	**.01**
Perceived performance	3.29 (.74)	3.01 (.99)	3.83 (.96)	**4.80 (.41)**	25.63^***^	.84	**.04**	**< .001**	**< .001**	**< .001**	**< .001**

*p < .05

**p < .01

***p < .001.

The values that reflect the best team functioning are presented in bold.

To provide more insight about the significant F-value obtained, Bonferroni post-hoc analysis revealed that teams with three task leaders scored significantly better on all the outcome variables (except for role clarity and social cohesion) compared to teams with two task leaders, teams with one task leader, and teams with no task leader. For some variables, also teams with two task leaders showed significantly better values than teams with one task leader (such as relationship conflict, collective efficacy, and perceived performance) or no task leaders (such as relationship conflict and perceived performance). To summarize, the results indicated that shared leadership within the role of task leadership (i.e., ideally having three task leaders) benefits team effectiveness the most, which confirms H1a.

#### Social leadership

Table 5 presents the different outcomes for each of the different leadership structures (i.e., when having zero, one, or two social leaders). The results revealed a significant difference between the three leadership structures with respect to role conflict, task conflict, social cohesion, and perceived performance (Wilk’s Λ = .84, F_(18,1026.00)_ = 5.36, *p* < .001; partial η^2^ = .09). Bonferroni post-hoc analysis showed that social cohesion in the team was only maximal when having two social leaders compared to having only one leader, thereby only partly supporting H1b. For other outcomes, such as role conflict, task conflict and perceived performance, having only one social leader seems to be the most favorable situation.

**Table 5:** Differences between zero, one, or two social leaders.

	0 leaders	1 leader	2 leaders	*F*	*Differences between number of leaders (p)*		
	*M (SD)*	*M (SD)*	*M (SD)*		0–1	0–2	1–2
All teams	(*n* = 121)	(*n* = 251)	(*n* = 159)				
Role clarity	7.38 (1.21)	**7.50 (1.27)**	7.34 (1.47)	.93	1.00	1.00	.61
Role conflict	2.30 (.88)	**2.00 (.79)**	2.30 (.85)	8.58^***^	**.004**	1.00	**.001**
Task conflict	3.36 (1.27)	**3.01 (1.31)**	3.43 (1.48)	5.46^**^	.07	1.00	**.007**
Relationship conflict	2.67 (1.34)	2.65 (1.42)	**2.50 (1.43)**	.74	1.00	.92	.84
Task cohesion	6.50 (1.58)	6.55 (1.72)	**6.72 (1.45)**	.76	1.00	.78	.95
Social cohesion	7.00 (1.58)	6.53 (1.75)	**7.29 (1.50)**	10.98^***^	**.09**	.43	**< .001**
Collective efficacy	3.43 (.66)	**3.57 (.71)**	3.49(.66)	1.89	.19	1.00	.66
Intention to continue	3.55 (.99)	3.70 (.98)	**3.80 (.89)**	2.29	.61	.09	.75
Perceived performance	3.60 (.97)	**3.67 (1.08)**	3.35(1.01)	4.68^**^	1.00	.16	**.008**
Male teams	(*n* = 53)	(*n* = 164)	(*n* = 100)				
Role clarity	**7.74 (1.03)**	7.50 (.39)	7.34 (.62)	1.42	.30	.42	1.00
Role conflict	2.25 (.91)	**2.04 (.80)**	2.30 (.81)	3.62^*^	.36	1.00	**.04**
Task conflict	3.31 (1.36)	**3.01 (1.35)**	3.46 (1.25)	3.84^*^	.45	1.00	**.02**
Relationship conflict	**2.01 (.98)**	2.73 (1.52)	2.56 (1.44)	5.20^**^	**.004**	.06	1.00
Task cohesion	**6.91 (1.37 )**	6.59 (1.72)	6.65 (1.41)	.85	.61	.93	1.00
Social cohesion	**7.56 (1.03)**	6.42 (1.73)	7.02 (1.47)	12.05^***^	**< .001**	.12	**.01**
Collective efficacy	**3.72 (.50)**	3.57 (.66)	3.53 (.59)	1.59	.49	.25	1.00
Intention to continue	3.60 (.97)	3.58 (.96)	**3.76 (.85)**	1.03	1.00	1.00	.48
Perceived performance	**3.89 (.95)**	3.74 (1.03)	3.22 (.97)	10.51^***^	1.00	**< .001**	**< .001**
Female teams	(*n* = 68)	(*n* = 87)	(*n* = 59)				
Role clarity	7.06 (1.28)	**7.70 (.98)**	7.23 (1.53)	5.24^**^	**.007**	1.00	.06
Role conflict	2.33 (87)	**1.92 (.75)**	2.30 (.94)	5.51^**^	**.007**	1.00	**.02**
Task conflict	3.38 (1.21)	**3.00 (1.25)**	3.37 (1.81)	1.76	.27	1.00	.40
Relationship conflict	3.22 (1.35)	2.52 (1.23)	**2.38 (1.43)**	7.48^**^	**.005**	**.002**	1.00
Task cohesion	6.16 (1.69)	6.48 (1.76)	**6.87 (1.52)**	2.74	.81	.07	.52
Social cohesion	6.57 (1.81)	6.73 (1.75)	**7.76 (1.50)**	9.13^***^	1.00	**< .001**	**.001**
Collective efficacy	3.21 (.68)	**3.58 (.78)**	3.41 (.77)	4.54^*^	**.01**	.37	.78
Intention to continue	3.51(1.02)	**3.91 (.97)**	3.90 (.97)	3.43^*^	**.04**	.08	1.00
Perceived performance	3.36 (.94)	3.54 (1.12)	**3.56 (1.05)**	.69	1.00	1.00	1.00

*p < .05

**p < .01

***p < .001.

The values that reflect the best team functioning are presented in bold.

#### External leadership

Table 6 presents the different outcomes for each of the different leadership structures (i.e., when having zero, one, or two external leaders). Significant differences were found between these different leadership structures with respect to their effect on social cohesion and intention to continue (Wilk’s Λ = .86, F_(22,1022.00)_ = 4.39, *p* < .001; partial η^2^ = .07). Specifically, Bonferroni post-hoc analysis revealed that teams with two external leaders obtained better values on social cohesion and intention to continue than teams having only one external leader. These findings confirm H1c in that having shared leadership within the external leadership role is the most favorable situation.

**Table 6:** Differences between zero, one, or two external leaders.

	0 leaders	1 leader	2 leader	*F*	*Differences between* *number of leaders (p)*		
	*M (SD)*	*M (SD)*	*M (SD)*		0–1	0–2	1–2
All teams	(*n* = 32)	(*n* = 161)	(*n* = 338)				
Role clarity	**7.60 (1.07)**	7.45 (1.28)	7.40 (1.36)	.44	1.00	1.00	1.00
Role conflict	**2.05 (.81)**	2.10 (.85)	2.19 (.84)	1.08	1.00	1.00	.61
Task conflict	2.90 (.98)	**3.19 (1.36)**	3.25 (1.40)	.92	.84	.54	1.00
Relationship conflict	2.76 (1.45)	2.62 (1.46)	**2.59 (1.37)**	.20	1.00	1.00	1.00
Task cohesion	6.47 (1.91)	6.39 (1.72)	**6.70 (1.52)**	2.10	1.00	1.00	.14
Social cohesion	6.49 (1.93)	6.33 (1.76)	**7.16 (1.53)**	14.99^***^	1.00	.08	**< .001**
Collective efficacy	3.50 (.52)	**3.59 (.73)**	3.48 (.67)	1.35	1.00	1.00	.32
Intention to continue	3.42 (.80)	3.38 (1.02)	**3.78 (.94)**	4.17^*^	1.00	.12	**.04**
Perceived performance	3.63 (.71)	**3.72 (1.12)**	3.55 (.98)	3.01	1.00	1.00	.05
Male teams	(*n* = 17)	(*n* = 126)	(*n* = 174)				
Role clarity	**7.62 (1.07)**	7.41 (1.07)	7.48 (1.39)	.21	1.00	1.00	1.00
Role conflict	**1.80 (.70)**	2.17 (.85)	2.18 (.82)	1.65	.22	.23	1.00
Task conflict	**2.90 (1.05)**	3.32 (1.37)	3.14 (1.34)	1.08	.68	1.00	.80
Relationship conflict	3.41 (1.47)	2.69 (1.52)	**2.37 (1.34)**	5.08^**^	.15	**.01**	.17
Task cohesion	6.42 (1.81)	6.39 (1.69)	**6.88 (1.42)**	3.62^*^	1.00	.76	**.02**
Social cohesion	6.46 (1.58)	6.28 (1.75)	**7.21 (1.37)**	13.31^***^	1.00	.17	**< .001**
Collective efficacy	3.60 (.49)	3.53 (.69)	**3.62 (.56)**	1.00	1.00	1.00	.64
Intention to continue	3.53 (.83)	3.39 (.96)	**3.83 (.87)**	8.90^***^	1.00	.57	**< .001**
Perceived performance	3.35 (.61)	**3.70 (1.06)**	3.56 (1.04)	1.25	.52	1.00	.58
Female teams	(*n* = 15)	(*n* = 35)	(*n* = 164)				
Role clarity	7.58 (1.11)	**7.63 (.98)**	7.31 (.32)	.94	1.00	1.00	.51
Role conflict	2.33 (.86)	**1.78 (.69)**	2.22 (.87)	3.75^*^	.10	1.00	**.02**
Task conflict	2.91 (.93)	**2.74 (1.26)**	3.36 (1.46)	3.33	1.00	.69	.06
Relationship conflict	**2.02 (1.03)**	2.34 (1.19)	2.83 (1.39)	3.83	1.00	.07	.16
Task cohesion	**6.53 (2.08)**	6.37 (1.86)	6.52 (1.61)	.13	1.00	1.00	1.00
Social cohesion	6.52 (2.32)	6.44 (1.82)	**7.10 (1.68)**	2.23	1.00	.66	.13
Collective efficacy	3.39 (.58)	**3.79 (.82)**	3.33 (.75)	6.57^**^	.26	1.00	**.005**
Intention to continue	3.29 (.79)	**4.27 (.95)**	3.72 (.99)	6.11^**^	.004	.30	**.01**
Perceived performance	**3.93 (.70)**	3.74 (1.34)	3.40 (1.00)	3.41	1.00	.17	.23

*p < .05

**p < .01

***p < .001.

The values that reflect the best team functioning are presented in bold.

### Shared leadership across the different leadership roles

While the previous analyses examined one specific leadership role at a time, we aim to provide deeper insight in this section by taking into account the leadership structure in all the leadership roles simultaneously. More specifically, we first examined whether it is more important to have shared leadership across all leadership roles, or whether it is more beneficial to have shared leadership in one leadership role, while having only one or zero leaders in the other roles. To examine this research question, we dichotomized the extent of shared leadership as low (zero or one leaders) or high (two or three leaders). Next, we differentiated all possible combinations across the three leadership roles. In addition, we also added a balanced category, in which teams showed a balance in the number of leaders in each of the leadership role (i.e., either zero leaders across all leadership roles, one leader, or two leaders). A MANOVA revealed significant differences between the different leadership structures for all outcomes (Wilk’s Λ = .58, F_(54,2599.99)_ = 5.48, *p* < .001; partial η^2^ = .09; see Table 7). Bonferroni post-hoc analysis revealed that teams with a high number of task, social, and external leaders (HHH) scored significantly higher than other teams on role clarity, task cohesion, collective efficacy, intention to continue, and perceived performance. Furthermore, the teams with a high number of leaders in all three roles (HHH) also showed significantly less role conflict (compared to LHH and Balanced groups) and less task and relationship conflict (compared to all other groups). The results thus reveal that, in contrast to H2, it is not a balanced number of leaders in each role, but rather a high degree of shared leadership across and within the different leadership roles that yields the most benefits for team functioning and team effectiveness.

**Table 7:** Variables scores for athlete leaders groups.

	LLL (a)	HHH (b)	HLL (c)	LHH (d)	LLH (e)	HLH (f)	Balanced	*F*
	*M (SD)*	*M(SD)*	*M (SD)*	*M (SD)*	*M (SD)*	*M (SD)*	*M (SD)*	
All teams	(*n* = 56)	(*n* = 20)	(*n* = 84)	(*n* = 77)	(*n* = 148)	(*n* = 31)	(*n* = 115)	
Role clarity	7.72 (1.22)	**8.16 (.67)**	7.46 (1.23)	7.07 (1.62)^b^	7.48 (1.14)	7.28 (1.72)	7.32 (1.32)	2.67^*^
Role conflict	2.01 (.88)	**1.57 (.52)**	2.12 (.83)	2.45 (.77)^b^	2.12 (.85)	2.04 (.73)	2.25 (.87)^b^	3.95^**^
Task conflict	2.80 (1.20)	**1.32 (.38)**^a^	3.15 (1.31)^b^	3.77 (1.35)^abc^	3.10 (1.35)^abd^	3.08 (1.15)^b^	3.60 (1.33)^abe^	12.62^***^
Relationship conflict	2.46 (1.24)	**1.17 (.28)**^a^	2.22 (1.08)^b^	3.24 (1.47)^abc^	2.63 (1.34)^bd^	2.94 (1.26)^b^	2.71 (1.62)^b^	8.22^***^
Task cohesion	6.97 (1.50)	**8.13 (.79)**	6.68 (1.53)^b^	6.34 (1.51)^b^	6.67 (1.64)^b^	6.71 (1.36)^b^	6.11 (1.75)^ab^	6.01^***^
Social cohesion	6.97 (1.41)	**7.60 (1.28)**	6.43 (1.75)	7.01 (1.63)	7.18 (1.54)^c^	6.37 (1.45)	6.63 (1.90)	3.52^**^
Collective efficacy	3.80 (.47)	**4.22 (.41)**	3.67 (.65)^b^	3.29 (.61)^abc^	3.42 (.68)^ab^	3.75 (.67)^de^	3.36 (.72)^abcf^	10.86^***^
Intention to continue	3.64 (.91)	**4.68 (.46)**^a^	3.66 (1.03)^b^	3.58 (.91)^b^	3.80 (.98)^b^	3.55 (.94)^b^	3.57 (.91)^b^	4.68^***^
Perceived performance	3.95 (.85)	**4.80 (.41)**^a^	4.16 (.69)^b^	2.80 (.82)^abc^	3.55 (1.07)^abcd^	3.74 (.77)^bd^	3.18 (1.03)^abcef^	26.46^***^
Male Teams	(*n* = 56)	(*n* = 0)	(*n* = 48)	(*n* = 58)	(*n* = 58)	(*n* = 16)	(*n* = 81)	
Role clarity	7.72 (1.23)		7.30 (1.37)	7.43 (1.44)	**7.85 (.86)**	6.59 (2.10)^ae^	7.26 (1.40)	3.28^**^
Role conflict	2.01(.89)		2.30 (.81)	2.29 (.76)	**2.00 (.82)**	2.08 (.82)	2.22 (.86)	1.47
Task conflict	2.80 (1.20)		3.69 (1.26)^a^	3.36 (1.19)	**2.60 (1.38)**^**cd**^	3.15 (1.05)	3.51 (1.38)^ae^	6.18^***^
Relationship conflict	2.46 (1.24)		2.51 (1.17)	3.09 (1.48)	**1.86 (.93)**^**d**^	3.04 (1.38)^e^	2.67 (1.75)^e^	5.26^***^
Task cohesion	6.97 (1.49)		6.37 (1.41)	6.46 (1.49)	**7.37 (1.27)**^**cd**^	6.54 (1.56)	6.28 (1.79)^e^	4.59^***^
Social cohesion	6.99 (1.41)		6.19 (1.62)	6.93 (1.53)	**7.74 (.97)**^**e**^	6.48 (1.41)	6.35 (1.86)^e^	7.74^***^
Collective efficacy	**3.82 (.49)**		3.53 (.63)	3.40 (.55)^a^	3.78 (.49)^d^	3.64 (.53)	3.44 (.74)^ae^	5.07^***^
Intention to continue	3.65 (.91)		3.29 (.95)	3.72 (.94)	**4.05 (.88)**^**c**^	3.54 (.91)	3.51 (.87)^e^	4.26^**^
Perceived performance	3.96 (.85)		4.00 (.65)	2.72 (.83)^ac^	**4.10 (1.00)**^**d**^	3.63 (.72)^d^	3.41 (1.08)^acde^	18.62^***^
Female Teams	(*n* = 0)	(*n* = 20)	(*n* = 36)	(*n* = 19)	(*n* = 90)	(*n* = 15)	(*n* = 34)	
Role clarity		**8.16 (.67)**	7.67 (1.02)	5.99 (1.68)^bc^	7.24 (1.23)^bd^	8.02 (.69)^d^	7.47 (1.13)^d^	8.97^***^
Role conflict		**1.57 (.52)**	1.88 (.81)	2.92 (.61)^bc^	2.20 (.86)	2.00 (.65)	2.31 (.91)^b^	6.87^***^
Task conflict		**1.32 (.38)**	2.43 (.98)^b^	5.04 (.96)^bc^	3.42 (1.23)^bcd^	3.00 (1.27)^bd^	3.79 (1.16)^bdd^	28.43^***^
Relationship conflict		**1.17 (.28)**	1.83 (.77)	3.65 (1.36)^bc^	3.13 (1.33)^bc^	2.82 (1.15)^b^	2.77 (1.25)^bc^	15.87^***^
Task cohesion		**8.13 (.79)**	7.10 (1.62)	6.04 (1.54)^b^	6.23 (1.68)^b^	6.89 (1.14)	5.69 (1.60)^bc^	8.40^***^
Social cohesion		**7.60 (1.28)**	6.81 (1.86)	7.32 (1.93)	6.81 (1.71)	6.26 (1.54)	7.20 (1.97)	1.49
Collective efficacy		**4.22 (.41)**	3.86 (.64)	2.93 (.65)^bc^	3.18 (.68)^bc^	3.88 (.80)^de^	3.12 (.64)^bcf^	16.44^***^
Intention to continue		**4.68 (.46)**	4.15 (.95)	3.12 (.65)^bc^	3.64 (1.01)^b^	3.56 (1.01)^b^	3.71 (1.02)^b^	7.37^***^
Perceived performance		**4.80 (.41)**	4.39 (.69)	3.05 (.71)^bc^	3.21 (.95)^bc^	3.87 (.83)^b^	2.59 (.66)^bcef^	32.31^***^

*p < .05

**p < .01

***p < .001.

The order of the letters refers to task, social, and external leadership, respectively. Groups’ interpretation notation: L = Low (zero and one leader), H = High (two and three leaders). LLL (a) = Low Task, Low Social, Low External; HHH (b) = High Task, High Social, High External; HLL (c) = High Task, Low Social, Low External; LHH (d) = Low Task, High Social, High External; LLH (e) = Low Task, Low Social, High External; HLH (f) = High Task, Low Social, High External; Balanced = equal number of leaders on each of the three roles. The values that reflect the best team functioning are presented in bold. The results of the Bonferroni post-hoc analysis are indicated with the superscripts a-f, which reflect the significant difference between the different leadership structures.

### Differences between male and female teams

#### Most common leadership structure

For both male and female teams, the majority of the teams had one task leader, one social leader, and two external leaders (Table 1).

#### Most effective leadership structure

To identify potential differences between male and female teams in the effectiveness of the different leadership structures, we conducted MANOVAs. The first step was to run a two-way MANOVA to examine whether there were differences in players’ perception of team effectiveness based on the interactions between gender and the different leadership roles. Significant effects were observed for the interactions between gender and task leadership (Wilk’s Λ = .64, F_(54,2599.99)_ = 4.36, *p* = .<001; partial η^2^ = .07), social leadership (Wilk’s Λ = .69, F_(45,2284.45)_ = 4.35, *p* = .<001; partial η^2^ = .07), and external leadership (Wilk’s Λ = .72, F_(45,2284.46)_ = 3.88, *p* = .<001; partial η^2^ = .06). The second step was run a MANOVA for male and female teams separately. The effectiveness of the task, social, and external leadership structures for male and female teams are presented in Tables 4, 5 and 6, respectively.

#### Task leaders

First, regarding the number of task leaders, our analyses yielded significant differences between the different leadership structures both in male (Wilk’s Λ = .78, F_(18,608.00)_ = 4.48, *p* < .001; partial η^2^ = .12) and female teams (Wilk’s Λ = .55, F_(27,575.98)_ = 4.76, *p* < .001; partial η^2^ = .18). More specifically, both in male and female teams, teams with a high number of task leaders showed the highest team effectiveness. More specifically, for female teams, Bonferroni post-hoc analysis revealed that teams with three task leaders scored significantly better on all the outcome variables compared to teams with two task leaders (except for role clarity, role conflict and social cohesion), compared to teams with one leader (except for social cohesion), and teams with no task leaders (except for social cohesion). Likewise, in male teams, teams with two task leaders perceived their performance to be better than that of teams with one or zero leaders.

#### Social leaders

Second, regarding the number of social leaders, the results revealed significant differences for female (Wilk’s Λ = .71, F_(18,608.00)_ = 6.38, *p* < .001; partial η^2^ = .16) and male teams (Wilk’s Λ = .74, F_(18, 396.00)_ = 3.55, *p* < .001; partial η^2^ = .14). For female teams, it was better to have one or two social leaders; in contrast, male teams showed higher team effectiveness when having one or zero social leaders. Bonferroni post-hoc analysis revealed that female teams with two social leaders showed a stronger social cohesion compared with teams with one or zero leaders, and less relationship conflict compared with teams with zero leaders. In contrast, in male teams, only social cohesion was reported to be significantly better in teams with two social leaders compared to teams with one social leader.

#### External leaders

Third, regarding the most optimal number of external leaders, our findings yielded significant differences for female (Wilk’s Λ = .71, F_(18,396.00)_ = 4.02, *p* < .001; partial η^2^ = .15) and male teams (Wilk’s Λ = .78, F_(18,608.00)_ = 4.43, *p* < .001; partial η^2^ = .12). Male teams achieved better team effectiveness when having two external leaders, whereas female teams obtained better results with only one external leader. Specifically, Bonferroni post-hoc analyses revealed that male teams with two external leaders reported less relationship conflict compared to teams with zero leaders, and higher task cohesion, social cohesion, and intention to continue compared to teams having only one external leader. In contrast, female teams with two external leaders reported higher role conflict and lower collective efficacy and intention to continue than teams having only one external leader.

## Discussion

In the present study, our aim was three-fold, namely (1) to identify the number of task, social, and external leaders in sports teams; (2) to examine the effectiveness of different leadership structures; and (3) to examine possible differences between male and female teams.

### Most common leadership structure

First, based on Kogler Hill [38] and Eys et al. [20], we differentiated between task, social, and external leadership. For each of these leadership roles, we analyzed the number of leaders (i.e., players who were endorsed as a leader by at least half of their team members in that specific leadership role), thereby differentiating between zero leaders, one leader, and two or three leaders, where the latter two refer to shared leadership within a specific leadership role. The results of the present study showed clear differences between the teams with respect to their leadership structure.

The most common structure within the teams was to have one task leader, one social leader, and two external leaders. The results with respect to task and social leadership are in line with earlier findings by Fransen [19]. However, the higher number of external leaders clearly contrasts with earlier research that reported a lower number of external leaders [8, 19, 20]. An important difference in research design may have accounted for this difference, as the present study relied on a data collection in professional soccer teams, whereas the other studies were carried out with college or university athletes [8, 20] or competitive athletes from lower competition levels [19] in a variety of team sports. Within the present elite sporting context, external leaders may be more prominent, as more contact with media and sponsors is needed at this level. We can conclude that most teams show a structure of shared leadership across the different leadership roles, with each role being occupied by at least one player. Only with respect to external leadership, teams also showed shared leadership *within* this leadership role. Further research in different age groups or competition levels is necessary to test the generalizability of our findings.

### Most effective leadership structure

To analyze the optimal leadership structure in sports teams, we started by examining each of the leadership roles and investigating the ideal number of leaders within each leadership role. Next, we examined the ideal number of leaders across the different leadership roles.

#### Ideal leadership structure *within* each leadership role

Regarding the second purpose of the study, it was hypothesized that teams in which multiple leaders take the lead in a particular leadership role would score better on each of the outcome variables than teams in which a single or zero leaders covered that leadership role. The results of the present study supported our hypothesis for most indicators of team effectiveness included in this study. More specifically, teams with a structure of three task leaders reported higher task cohesion, collective efficacy, intention to continue and perceived performance, while showing less role conflict, task conflict, and relationship conflict. Likewise, teams with three task leaders also reported the highest role clarity and the strongest social cohesion, although the difference with other leadership structures was not significant.

In general, we can conclude that having three task leaders is the most effective leadership structure for professional soccer teams. Several reasons potentially underpin this finding. First, the knowledge and expertise of three task leaders exceeds the capacity of a single individual [2, 18]. Second, the presence of multiple task leaders allows sharing the burden of leadership [6, 19]. Indeed, instead of one task leader having to provide directions to all of his/her team members, multiple leaders can more easily cover the entire soccer field. This becomes especially important in soccer, where players are positioned relatively far from each other compared to other sports. In this case, having only one source of communication would seriously hamper the team’s communication and functioning. Third, when having multiple task leaders on the team, leadership continuity is more likely [6]. Having only one task leader entails considerable risks because, if that task leader is not playing due to an injury or sanction or he or she leaves for another team, the team suddenly lacks task leadership, which can seriously hamper the team’s effectiveness.

With respect to social leadership, teams with two social leaders (compared to having one or no social leaders) reported stronger social cohesion. Furthermore, shared leadership within the social leadership role tends to be related to more task cohesion and less relationship conflict. These findings are in line with previous research revealing that the more social leadership is shared between the players, the higher the team's social connectedness [51], as well as its social and task cohesion [19]. Another benefit of having multiple social leaders is that players can choose which of these leaders they trust the most to discuss personal problems [19]. Also, if conflict arises in the team, different players can step up depending on the nature of the conflict or the players involved, which maximizes the effectiveness of the intervention.

Similarly, teams having shared leadership within the external leadership role (i.e., having two external leaders instead of one or zero) reported higher social cohesion and stronger intention to continue, again in line with the findings of Fransen [19] showing the strongest density of the social cohesion network in teams with two leaders.

In sum, we can conclude that shared leadership within each leadership role is beneficial for the team’s effectiveness. This finding is aligned with previous research showing that having multiple leaders in a leadership role is related to a higher team cohesion, stronger collective efficacy beliefs, and higher team identification [15]. Furthermore, the results of this study also went beyond the scope of previous studies by including indicators of detrimental team functioning, such as role conflict and team conflict. Given the scarcity of research examining athlete leadership roles and their association with negative outcomes, these findings extend knowledge about leadership roles and their relationship with positive and negative indicators of team functioning.

#### Ideal leadership structure *across* the three leadership roles

After identifying the ideal leadership structure within each leadership role, the next aim is to bring these findings together and investigate the ideal leadership structure across all leadership roles. In fact having a high number of leaders in one leadership role may imply that it would be better to have fewer leaders in the other roles. To provide an answer to this question, we created several combinations of the leadership structures in the three roles, depending on the existence of either a high extent of shared leadership (two or three leaders) or a low extent of shared leadership (zero or one leader) in each of the roles.

In general, the results showed that the teams that had a high number of leaders in each of the roles showed greater team effectiveness. More specifically, these teams reported higher role clarity, stronger task cohesion, collective efficacy, intention to continue, and better perceived performance, while reporting less role conflict, task conflict, and relationship conflict compared to other teams. These results contrast with earlier findings of Eys et al. [20], who found that teams that perceived a similar number of task, social, and external leaders on their teams (regardless of whether this number was high or low) were more satisfied with their team’s performance and team cohesion. Our study went more into detail and showed that, for every leadership role separately, having more than one leader may be preferable to having one or zero leaders. Therefore, it seems logical that the advantages of having a shared leadership structure within each leadership role (i.e., sharing the burden, more commitment, and higher identification) also holds for the structure across the three leadership roles.

The present findings lead to two important conclusions. First, each of these leadership roles is essential for the team’s functioning, and having leaders in each of these roles is clearly better for the team’s effectiveness than having zero leaders. These results are in line with previous research that linked the fulfillment of these different leadership roles to outcomes such as team identification, team confidence, shared purpose, goal commitment, and, ultimately, also to measures of perceived and objective performance [12, 17].

Second, the findings reveal that it is not only important to have leaders occupy the different leadership roles, but it is also important to share leadership responsibilities about a specific role with more than one player. In other words, coaches should strive for a structure of shared leadership, not only across, but also within each leadership role [12, 15]. It is important to note that the maximum number of observed leaders within a team was limited to two or three, depending on the role. This means that the results do not imply that every player in the team should adopt a leadership role. Instead, our findings argue for a hybrid model of shared leadership, with the leadership responsibilities for every role shared among a limited number of players. The results are in line with previous research [19] suggesting that, on the one hand, the greater the number of leaders in each leadership role, the more leadership qualities the team will have, which will lead to more task, social, and external support for the other team members. On the other hand, having more leaders might also hinder role clarity and lead to role conflict, as “too many cooks might spoil the broth”. Therefore, authors argue for a structure in which a limited leadership group takes the lead, an argument that the present study can corroborate.

### Differences between male and female teams

For both male and female teams, shared leadership across and within leadership roles was seen as the most effective leadership structure. Apart from these general similarities, some differences between male and female teams could also be noted. With respect to task leadership, female teams obtained the best scores on all variables when having three task leaders. For male teams, one task leader seemed more beneficial to avoid task conflict, while teams with two social leaders reported greater social cohesion but lower levels of role conflict, task conflict, and perceived performance. Likewise, female teams with two social leaders presented stronger social cohesion and less relationship conflict, but more role conflict. Finally, with respect to external leadership, male teams with shared leadership (two leaders) reported greater benefits than teams with any other structure (zero or one leader), but female teams with two external leaders reported higher role conflict and lower collective efficacy and intention to continue. These results seem to suggest that male teams show more benefits when there are more task and external leaders, whereas female teams reveal more benefits when there are more task and social leaders. The gender differences are consistent with other group variables such as cohesion, where there is evidence that the cohesion-performance relationship does differ between males and females [22, 24]. Therefore, coaches who want to implement a shared leadership structure in their teams should take their players’ gender into account when deciding on the number of leaders to appoint. The observed differences should encourage future research on gender differences to consolidate these findings.

### Practical implications

The present study contributes to the growing body of knowledge regarding the leadership structure in sports teams. From an applied perspective, the findings provide more insight about the optimal number of leaders for team effectiveness and emphasize the importance of shared leadership structures. Therefore, coaches can be recommended to implement a structure of shared leadership in their teams by appointing the adequate number of athlete leaders in each of the leadership roles (i.e., as established in the current study). This is particularly important given the fact that our study findings revealed that teams in which the leadership responsibilities rest solely on the shoulders of the coach, and no athlete leaders were present, had poorer performance than teams in which the leadership was shared.

The present findings help the coaches to identify the adequate number of athlete leaders, depending on the gender of the team (e.g., male teams have ideally more external leaders than female teams, whereas the opposite is true for social leaders). Of course, identifying the appropriate number of athlete leaders is only a part of the entire process of setting up an effective structure of shared leadership [51, 52]. Another two elements are essential for coaches in this process: (1) selecting the right athletes for the athlete leadership job; and (2) further developing their leadership qualities [51, 52]. We will discuss each of these elements, so that, together with identifying the adequate number of athlete leaders (as a result of the present study), they provide coaches with a full insight about how to set up an effective structure of shared leadership.

The first challenge to tackle here is how to identify the right athletes for the job. That is not always straightforward. Although coaches often think they have adequate insight about the leadership potential of their athletes, their insight often contrasts with the perceptions of their athletes. As an illustration, research revealed that only in 1% of the teams, were team captains (often appointed by the coach) perceived as the best leaders by their teammates [15]. When the appointed captains are not perceived as good leaders by their teammates, their leadership is not likely to be effective [10]. Therefore, it is important for a coach to consider this choice well.

In this vein, Cotterill and Fransen [6] carried out a review that revealed some key characteristics of athlete leaders. Some examples are ambition, competitiveness, responsibility, seniority in the team, team tenure, skill level, and often, these leaders also occupy a central position [6, 8, 36]. However, the most important aspect identified was for leaders to be accepted by their teammates; in other words, to have a large support base in the team [6, 51, 53]. This latter aspect is the key assumption in the Shared Leadership Mapping process, developed by Fransen et al. [54]. This process is based on the perceptions of the team members (rather than on the coach’s perception) and uses social network analysis to map leadership perceptions in the team. The resulting network clearly identifies the best leaders in a specific role (i.e., those who are perceived as best leaders by their teammates) at the center of the network. Hence, the coach has all the information not only to appoint the adequate number of leaders (based on our study findings) but also to identify the suitable athletes to fulfill the job. As the process ensures that these leaders have a large support base in their team, their leadership has the highest chance of being successful.

Once these leaders have been appointed, and an effective structure of shared leadership has thus been implemented, the second challenge to tackle is how to further improve the leadership potential of the appointed leaders. One of the latest trends in leadership research, based on the social identity approach to leadership [53], emphasizes the importance of a leader's ability to build a shared identity within the team; in other words, a shared feeling of “we” and”us” [52, 55]. Fransen et al. [52] and Slater et al. [55] relied on these principles to design interventions aimed at strengthening these identity leadership competencies in the appointed athlete leader. More specifically, in the 5R^S^ Shared Leadership Program of Fransen et al. [52], based on Shared Leadership Mapping (as explained above), the appointed leaders are taught how to create, embody, advance, and embed a collective sense of ‘us’ in their teams. To achieve this aim, the appointed athlete leaders, together with their teams, are guided through five phases, each focusing on one core question; (1) the Readying phase (Why does “we” matter?–A general introduction session); (2) the Reflecting phase (Who are we?–Identifying the team’s core values); (3) the Representing phase (What do we want to be?–Identifying the team’s aspirations); (4) the Realizing phase (How do we become what we want to be?–Implementing the strategies to achieve the team goals); and (5) the Reporting phase (Are we becoming what we want to be?–Monitoring progress towards team goals). Despite the initial validation of the effectiveness of these intervention programs, more research is required to explore its generalizability in different sports, at different competitive levels, and in different cultures.

### Strengths, limitations, and future research avenues

We can point out four important strengths of our study. First, this is one of the first studies that examines the number of athlete leaders on the team and, more importantly, relates that number of leaders to the team’s effectiveness in order to establish which structures are the most beneficial. Second, in contrast to previous research, we did not only include positive indicators of team effectiveness (e.g., performance), but we also assessed possible barriers to effective team functioning (e.g., team conflict). As such, we could obtain a deeper insight about the positive—and potentially also the negative—outcomes of a specific leadership structure. Third, the study is carried out with a large sample of high-level professional players, participants that are difficult to access but who provide very useful information on what goes on in high-performance sports teams. Finally, it should be noted that evaluation of athlete leadership was based on the perceptions of all the players, which is more reliable than methods such as self-reports or a single focus on the team captains.

With regard to the limitations, first, the findings obtained were correlational and cross-sectional, so no causal inferences can be made about the relationships between athlete leadership and the various outcome variables. Nevertheless, our results are consistent with theoretical predictions and previous empirical research concerning the association between athlete leadership and indicators of team effectiveness [15, 19]. Studies adopting an experimental design could provide more insight about the causal impact of the number of athlete leaders on the outcome variables. Furthermore, longitudinal studies could provide more information about the stability of the number of leaders in the course of a season. Given that other studies have demonstrated changes in group processes over time [29, 33, 56], the same may hold for leadership structures.

Second, we measured the number of leaders within the teams, but not the quality of their leadership. Several studies have shown that the quality of leadership is essential for a team’s effectiveness [17, 51]. For example, Fransen et al. [12] showed that, in professional rugby teams, the quality of the leadership in every leadership role was significantly related to team confidence, shared purpose, goal commitment, team climate, and both perceived and objective performance. In this regard, teams with one great leader may show better team effectiveness than teams with three poor leaders. Therefore, future studies should take into account the quality of the leaders when analyzing the ideal leadership structure [8, 9, 51].

Third, this study employed three types of leadership roles that had originally been established in the early studies [8, 20]. However, Fransen et al. [15] have recently found empirical evidence of a fourth role, namely motivational leadership. Later studies have also established the importance of this leadership role for team effectiveness and, more specifically, for task and social cohesion [11], team confidence, and team identification [17], and also for goal commitment, team climate, and both perceived and objective performance [12]. It is noteworthy is that these studies also indicate the importance of shared leadership within this motivational leadership role (i.e., benefits of having more than one motivational leader).

Finally, the generalization of our findings to other population samples and sports should be done with caution because our sample comprised only players from a particular sport (i.e., soccer), from a particular level (i.e. professional), and from a particular country (i.e., Spain). Furthermore, the different competition levels used in the present study for female teams (i.e., highest level) and for male teams (i.e., third level) could confound the generalization of our findings. It should be noted, however, that the level of professionalism, and economic and social recognition were very similar in both levels of competition. Further research in different sports and competition levels is necessary to confirm the generalizability of our findings.

Despite the aforementioned limitations, we believe that this work makes a unique contribution to the literature by examining the structure of athlete leadership and its relationship with a variety of important indicators of team effectiveness in professional sport. The findings corroborate the recent evolution from more hierarchical leadership models towards the idea of shared leadership, in which multiple players are sharing leadership responsibilities. Thus, it can be concluded that by implementing a structure of shared leadership not only across, but also within the different leadership roles, coaches can lead their team towards success.

## Supporting information

S1 File Measure3_Leadership.sav. (SAV)
